# Identification of Peroxiredoxin 1 as a novel interaction partner for the lifespan regulator protein p66Shc

**DOI:** 10.18632/aging.100017

**Published:** 2009-01-30

**Authors:** Melanie Gertz, Frank Fischer, Martina Leipelt, Dirk Wolters, Clemens Steegborn

**Affiliations:** ^1^ Department of Physiological Chemistry, Ruhr-University Bochum, 44801 Bochum, Germany; ^2^ Department of Analytical Chemistry, Ruhr-University Bochum, 44801 Bochum, Germany

**Keywords:** apoptosis, mitochondria, Prx1, reactive oxygen species, ROS

## Abstract

Damage caused by reactive oxygen species (ROS) contributes to many aging processes and accompanying diseases. ROS are toxic side products of cellular respiration, but also function as signal, e.g. in the mitochondrial apoptosis pathway. The protein p66Shc, which has been implicated in life-span regulation and aging-related diseases, is a central player in stress-induced apoptosis and the associated ROS burst. Stress signals, such as UV radiation or ROS themselves, activate p66Shc, which was proposed to stimulate its H_2_O_2_ forming activity, ultimately triggering mitochondrial disintegration. However, mechanistic details of H_2_O_2_ formation and apoptosis induction by p66Shc and regulation of these activities remain to be revealed. Here, we describe the effects of Ser36 phosphorylation and Pin1 binding on p66Shc activity, and the identification of Peroxiredoxin 1 (Prx1) as a novel interaction partner for the unique p66Shc N-terminal domain. Prx1 was identified in affinity experiments as dominant interaction partner. Complex formation leads to disassembly of Prx1 decamers, which is known to increase its peroxidase activity. The interaction leads to reduction of the p66CH2CB tetramer, which reduces its ability to induce mitochondrial rupture. Our results indicate that p66CH2CB and Prx1 form a stress-sensing complex that keeps p66Shc inactive at moderate stress levels.

## Introduction

Aging is usually accompanied by diseases like cardiovascular dysfunctions or cancer [1], indicating that they are caused by related molecular processes [2-4]. A mechanism recognized to contribute to many aging processes and accompanying diseases is the accumulation of damage caused by reactive oxygen species (ROS) [5,6], which are mainly formed by the mitochondrial respiratory chain. Aging tissues exhibit elevated levels of oxidized lipids and proteins  [5]  and show increased expression of stress response genes [7]. Consistently, lifespan extension through Sirtuin activation or caloric restriction appears to be due to an increase of stress resistance and the alleviation of diseases [6,8,9]*.*

Under normal conditions, overall cellular ROS levels are kept low by antioxidants, such as glutathione (GSH). Besides their destructive role, however, ROS also act as signaling mediators [10,11]. They can,  e.g., initiate apoptosis by inducing permeability transition of mitochondrial membranes [12], resulting in cytochrome c (Cyt c) release and apoptosome activation [13]. ROS can represent oxidative stress upstream of this step, but cellular ROS levels can also be actively increased after pro-apoptotic stimuli [6]. ROS can thus establish a positive feedback loop, as described for the oxidant-induced activation of p53 that leads to ROS formation [6,14,15]. Consistently, antioxidant treatment protects cells from apoptosis not only if initiated by oxidants, but also if initiated by a wide variety of stressors and stimuli [16].

p66Shc appears to be a central player in stress-induced apoptosis and ROS amplification, in diseases caused by dysfunction of this system, and in life-span regulation [17,18]. Knocking out the shcA (Src homologous and collagen A) gene coding for p66Shc results in decreased ROS levels and a 30 % extended lifespan of rodents [19]. p66Shc has been implicated in promoting cancer cell growth and in aging-associated arterial dysfunctions [2,20], and has thus been suggested as a novel drug target [21]. p66Shc is the largest polypeptide encoded by the shcA locus, besides p46Shc and p52Shc. All isoforms share a phosphotyrosine-binding (PTB), a collagen homology (CH), and a Src homology 2 (SH2) domain; p52Shc shares an additional Cytochrome c binding domain (CB) with p66Shc, which carries a unique CH2 domain at its N-terminus. The mainly cytosolic isoforms p46Shc and p52Shc act as adaptor proteins regulating, e.g., elements of the Ras signaling pathway [18]. In contrast, p66Shc is located in the cytosol and the mitochondrial intermembrane space (IMS) [22-24]. Upon induction by stress factors, expression of the p66Shc protein increases [25], existing p66Shc is stabilized [26], and cytosolic p66Shc gets phosphorylated by protein kinase Cβ (PKCβ, followed by an interaction with the prolyl isomerase Pin1 and translocation to the IMS [24]. In this study, p66Shc has been suggested to directly form H_2_O_2_ [22], which is assumed to activate the permeability transition pore (PTP), ultimately causing rupture of mitochondria. Consistently, overexpression of p66Shc increases ROS level [27] and p66Shc-/- fibroblasts are resistant to apoptosis induced by several stressors [19,28,29]. Thus, p66Shc acts as sensor for ROS, but also for other stress factors, and on the other hand increases ROS formation leading to apoptosis initiation, presumably under overwhelming stress when repair systems cannot cope with the ROS anymore. However, the N-terminal p66Shc domain responsible for apoptosis induction and H_2_O_2_ formation could be shown to be a better apoptosis inducer in an oxidized, tetrameric form, but to produce more ROS in its reduced, dimeric form [30], indicating that the molecular details of p66Shc-mediated apoptosis are still not fully understood.

We showed previously that p66Shc can interact with thioredoxins (Trx), which reduce and thereby inactivate the N-terminal domains, CH2 and CB (p66CH2CB), that carry the apoptosis inducing and H_2_O_2_-forming activity of p66Shc [30]. Another prominent family of proteins contributing to redox metabolism and signaling are peroxiredoxins (Prx) [31]. Prx degrade H_2_O_2_ using a conserved cysteine residue. Mammals have six isoforms, Prx1-6, divided into classes depending on whether they exhibit just the catalytic (peroxidasic) cysteine or also an additional, resolving cysteine, and whether both cysteines are on the same polypeptide [32]. Prx are weak scavengers, but due to their abundance and broad substrate specificity they effectively degrade peroxides at low concentrations. Increased H_2_O_2_ levels, in contrast, inactivate Prx through overoxidation of the peroxidasic cysteine, making them ideal sensors for H_2_O_2_-mediated signaling [31]. Many Prx further exist in different oligomeric forms, a dimeric form with significant peroxidase activity, which was proposed to be stabilized by oxidation at the catalytic cysteine, and an oligomeric form with low peroxidase activity that appears to act as chaperone. Furthermore, Prx can interact with other proteins apparently for regulating their activity [31], such as the inhibitory interaction of Prx1 with the apoptosis signal-regulating kinase 1 (Ask1) [33].

Here, we describe the identification of Prx1 as a novel interaction partner for the unique N-terminal domain of p66Shc, and the biochemical characterization of this complex. Prx1 was identified in affinity experiments as dominant interaction partner. Complex formation leads to disassembly of the decameric form of Prx1, which is known to increase its peroxidase activity. The interaction leads to reduction of the p66CH2CB tetramer, which reduces its ability to induce mitochondrial rupture. These results indicate that p66CH2CB and Prx1 form a stress-sensing complex that keeps p66Shc inactive as long as stress levels remain moderate.

## Results

### p66Shc regulation through phosphorylation and interaction with Pin1

**Figure 1.**
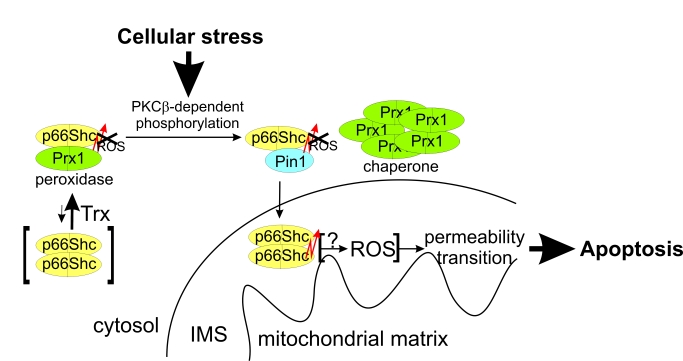
Effects of phosphorylation and Pin1 binding on p66Shc. **(a-c)** The p66CH2CB-dependent ROS-generation is enhanced by phosphorylation of Ser36 but inhibited in the presence of Pin1. Changes in fluorescence of 10 μM H_2_DFFDA were recorded after addition of 20 μM p66CH2CB WT **(a)** or the p66CH2CB-Ser36Asp mutant simulating Ser36 phosphorylation **(a-c)**, followed by 85 μM Na-dithionite (DN) and 50 μM CuSO_4_ (Cu) in the presence **(b+c)** or absence of Pin1 **(a-c)** and/or the dipeptide Ala-Pro **(c)**. **(d)** p66CH2CB-induced mitochondrial rupture is inhibited by phosphorylation of Ser36. Mitochondrial rupture was induced after addition of 7 μM CaCl_2_ by addition of 20 μM p66CH2CB WT or Ser36Asp and monitored photometrically. The initial sensitization with CaCl_2_ was omitted for clarity. **(e)** H_2_O_2_ oxidizes p66CH2CB. 10 μg p66CH2CB were incubated with 0.005 % H_2_O_2_ and subjected to non-reducing SDS-PAGE

Cells need to have mechanisms strictly silencing the cytotoxic activities of p66Shc, ROS-generation and apoptosis-induction, during cell cycle but stimulating them during cellular stress. PKCβ and the peptidyl-prolyl isomerase Pin1 have been reported to regulate the relocalization of p66Shc to mitochondria required for apoptosis initiation. PKCβ phosphorylates p66Shc at Ser36 in response to oxidative stress [19], which was reported already to increase cellular ROS levels [24]. We tested whether phosphorylation leads to a direct stimulation of the p66Shc-inherent oxidoreductase activity. We simulated phosphorylation by mutating Ser36 in  p66CH2CB to  Asp and compared the ROS-generating activity of the Ser36Asp mutant to wildtype (WT) protein in a fluorescence-based H_2_O_2_ assay (Figure 1a). The p66CH2CB-Ser36Asp mutant displayed a significantly increased activity compared to WT protein, independent of the dimer/tetramer equilibrium (data not shown) that is known to influence ROS formation [30], showing that phosphorylation at Ser36 indeed directly activates the oxidoreductase activity of p66CH2CB.

Phosphorylation of p66Shc activates ROS production but also induces an interaction with Pin1, assumed to lead to a peptidyl cis-trans isomerisation, which was reported to stimulate translocation of p66Shcinto the mitochondrial IMS [24]. We aimed to test whether the suggested peptide cis-trans isomerisation influences the ROS-forming activity of p66Shc. Indeed, addition of Pin1 to the fluorescence-based assay inhibited ROS-production by p66CH2CB-Ser36Asp, but only in a stoichiometry of about 1:1 p66CH2CB (Figure 1b). Catalytic Pin1 amounts (e.g. 1:100 Pin1:p66CH2CB-Ser36Asp) had no significant effect, suggesting that the interaction leads to complex formation rather than an enzymatic isomerisation of p66CH2CB-Ser36Asp. Consistently, addition of the dipeptide Ala-Pro, a known inhibitor of Pin1-induced isomerization, had no effect on the Pin1-dependent quenching of p66CH2CB-dependent ROS production (Figure 1c).

We next tested the effects of Ser36 phosphorylation and Pin1 binding in a mitochondrial swelling assay. Both oligomeric forms of p66CH2CB-Ser36Asp, dimer and tetramer, hardly induced rupture of mitochondria, in contrast to the active WT form, the tetramer (Figure 1d). This observation is consistent with the suggested necessity of Ser36 dephosphorylation, after interaction with Pin1, for enabling the proapoptotic function of p66Shc [24]. Adding Pin1 to p66CH2CB-Ser36Asp had no further effect on the already low mitochondria swelling activity of the mutant (data not shown). We conclude that phosphorylation at Ser36, which appears necessary for initiating relocation to the IMS [24], increases the ROS-generating activity of p66Shc. This apparently unintentional increase can be compensated in the cytosol by complex formation with Pin1. Once p66Shc reaches the IMS, however, dephosphorylation is required to enable mitochondrial rupture. These results reinforce the notion that the ROS-forming and apoptosis-inducing activities of p66Shc are not directly coupled, and that the physiological functions and regulation of these activities will have to be further studied for a complete understanding of this signaling system. H_2_O_2_ could, e.g., be an intrinsically formed p66Shc activator, as H_2_O_2_ can induce formation of oxidized p66Shc (Figure 1e), or H_2_O_2_ formation might just be a side reaction of a different redox reaction catalyzed by p66Shc (see below).

### p66CH2CB physically interacts with Prx1

**Figure 2.**
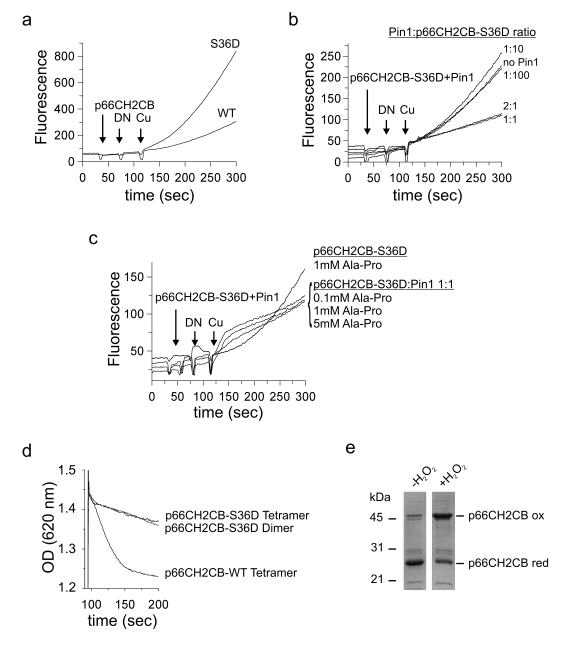
Identification of the novel p66Shc interaction partner Prx1. **(a)** *In vivo* pull down and subsequent MudPIT analyses identified Prx1 as an interaction partner of p66CH2CB. The proteins discussed in the text (Prx1, Prx3 and Hsp70) are highlighted in grey. **(b-d)** p66CH2CB interacts with dimeric and decameric Prx1 in a 1:1 stoichiometry and perturbs its decameric arrangement. 20 μg p66CH2CB WT **(b)** or p66CH2CB-Cys59Ser **(c)** were incubated with 40 μg Prx1 WT or 20 μg Prx1-Cys83Ser, respectively, for 1 hour at room temperature and subjected to BN-PAGE **(b+c)**. Lanes 1 and 2 from **(b)** are repeated in **(c)** for clarity. The p66CH2CB-Cys59Ser/Prx1-Cys83Ser interaction complex is marked by an arrow. **(d)** Bands labelled 1-7 in panel **(b)** were subjected to a second separation dimension, denaturing SDS-PAGE.

In order to identify novel interaction partners of p66Shc contributing to its complex regulation and localization, we performed an *in vivo* pull down experiment using immobilized p66CH2CB (a mixture of dimeric and tetrameric state) and cleared mitochondrial lysate. Due to the lack of hypothesis which protein might interact with p66CH2CB, we could not use specific antibodies to analyze the pull down eluate. Instead, we used a mass spectrometry-based multidimensional protein identification technology (MudPIT) [34] which is able to analyze complex protein solutions. In order to exclude non-specifically bound proteins, hits also found in control experiments without immobilized protein or with the unrelated protein Sirtuin 5 (Sirt5) were removed. Additionally, we used the number of spectrum counts (total number of successful MS/MS spectra) [35] as well as the sequence coverage of the detected proteins as parameters to semi quantify their abundance in the eluate. Interestingly, Prx1 stands out with a very high number of spectrum counts (122) and very high sequence coverage (55.8 %) besides proteins only identified with very low values (Figure 2a). Much weaker signals, but still specifically only in the p66CH2CB sample, were observed for the previously described p66Shc interaction partner Hsp70 [28] and for Prx3, which was identified with a sequence coverage of 8.9 % and 2 spectrum counts.

Peroxiredoxines are highly abundant in cells. However, in contrast to other abundant proteins, like glutamate-dehydrogenase 1 or catalase, which were found in all samples independent of presence and identity of an immobilized protein, Prx1 and Prx3 were exclusively detected in the p66CH2CB sample and not, e.g., in the Sirt5 control. To identify the p66Shc form which interacts with Prx1, we repeated the *in vivo* pull-down with the isolated dimeric state and the isolated tetrameric state of p66CH2CB. However, Prx1 was reproducibly found as dominant signal in both samples (data not shown) indicating that Prx1 might interact with both forms (see below). We conclude that Prx1 was strongly enriched, and possibly Prx3 to a lower extent, through a specific interaction with the immobilized p66CH2CB.

### p66Shc disassembles the Prx1 decamer

In order to confirm and to further characterize the complex of p66CH2CB and Prx1 we analyzed their interaction in BN-PAGE experiments using purified recombinant protein (Figure 2b, 2c). Prx1 is known to have two oligomeric states with different molecular functions: In its dimeric state it shows H_2_O_2_-degrading (peroxidase) activity, but switches to chaperone activity after formation of a decamer, consisting of five dimers, in response to heat stress or oxidative stress [36,37]. On the BN-gel WT Prx1 mainly exists as a decamer (Figure 2b, lane 1). However, in the presence of p66CH2CB the decameric Prx1 ring dissociates (Figure 2b, lane 4). The result is a ladder of bands  indicating  either active disassembly of Prx1 decamers by p66CH2CB or p66CH2CB-dependent stabilization of spontaneously disassembled Prx1 forms preventing instantaneous reassembly of the decamer.

The still not fully understood system of different oligomeric Prx forms, likely corresponding to different activities (dimer: peroxidase; decamer: chaperone) is influenced by two catalytic and one regulatory Cys residue. H_2_O_2_-degradation is accomplished through oxidation of the *peroxidasic* cysteine (Cys52 in mouse Prx1) by H_2_O_2_ into a sulphenic acid. This residue then forms an intermolecular disulfide with the *resolving* cysteine (Cys173 in mouse Prx1) of the second monomer within dimeric peroxiredoxins. Finally, Trx acts as an electron donor to recycle the system.

Likewise, p66CH2CB reversibly forms tetramers by disulfide bridging [30] and we observed that these disulfides can be exchanged with reduced Prx1 (see below), leading to disulfide-linked Prx1 monomers similarly to H_2_O_2_-dependent oxidation. The upper bands of the ladder in the BN-gel obtained in the presence of p66Shc might therefore be higher oligomeric complexes which are stabilized by disulfide-bridging of Prx1 molecules. In agreement, incubation of the decameric Prx1 with the p66CH2CB Cys59Ser mutant, which lacks the redox-active cysteine residue and therefore cannot oxidize Prx1, does not result in the ladder of bands. Instead, one dominant band above 100 kDa appears in the BN-gel (Figure 2c, lane 4). Furthermore, incubation of p66CH2CB WT or p66CH2CB-Cys59Ser with Prx1-Cys83Ser, a Cys83Ser regulatory mutant that cannot form higher Prx1 oligomers but exists only as a dimer, also results in this dominant band (Figure 2b, 2c, lane 5). This finding indicates that the Prx1 dimer might be the primary interaction partner for p66Shc.

The question arises whether the various bands all contain complexes or instead only consist of Prx1 oligomers. In order to identify the content of the bands, we separated their content in a second, denaturing gel electrophoresis dimension (Figure 2d). Bands 1-5 and 7 comprise both proteins, Prx1 as well as p66CH2CB, again proofing their interaction (Figure 2d, lane 1-5 and 7) and indicating that various complex oligomers can be formed. Band 6 of the Prx1 control, which has roughly the same size in the BN-gel as band 7 (Figure 2b), as expected only produced a Prx1 band in the denaturing gel (Figure 2d, lane 6). Further, the proteins in bands 1-3 and 7 appeared in about equal amounts on the SDS-gel, which indicates an interaction stoichiometry of 1:1. Contrary, bands 4 and 5 mainly consist of p66Shc (Figure 2d, lane 4 & 5). However, they are very close to the band of tetrameric p66Shc, which likely contaminates the complex bands and artificially influences the protein ratio. We conclude that p66CH2CB and Prx1 indeed interact physically, apparently in a stoichiometry of 1:1, and that this interaction promotes disassembly of the decameric (chaperone) form of Prx1. Our findings render the dimeric Prx1 form the most likely interaction partner of p66CH2CB. Furthermore, p66CH2CB/Prx1 complex formation does not dependent on disulfide-bridging between Prx1 and p66CH2CB, but disulfide-bonds between Prx1 monomers can lead to formation of higher oligomeric complexes.

### Functional characterization of the p66CH2CB/Prx1 complex

**Figure 3.**
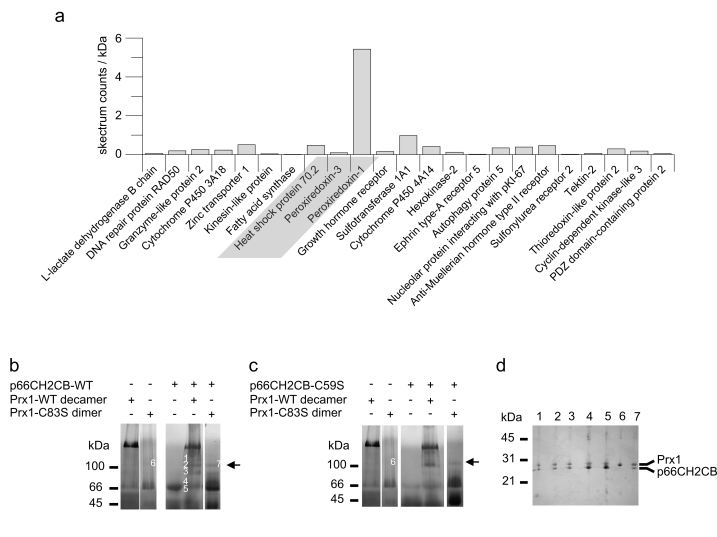
Functional characterization of the p66Shc/Prx1 interaction. **(a)** The dimer is the peroxidase-active form of Prx1. Changes in fluorescence of 10 μM H_2_DFFDA were recorded after addition of 0.005 % H_2_O_2_ in the absence and presence of 20 μM Prx1 WT or the mutants Prx1-Cys52Ser and Prx1-Cys83Ser. **(b)** p66CH2CB has an additional copper-independent activity which is inhibited by Prx1. Changes in fluorescence of 10 μM H_2_DFFDA were recorded after addition of 20 μM p66CH2CB and 0.005 % H_2_O_2_ in the absence and presence of 20 μM Prx1 WT or the mutants Prx1-Cys52Ser and Prx1-Cys83Ser. **(c)** p66CH2CB and Prx1 perform a disulfide exchange reaction with each other. 10 μg p66CH2CB were incubated with Prx1 for 1 hour at room temperature and subjected to non-reducing SDS-PAGE. Reduced p66CH2CB is formed, and the reduced Prx1 is concurrently oxidized. **(d)** Trx1 does not prevent formation of the major p66CH2CB/Prx1 complex, indicating separate binding sites for Prx1 and Trx1. 15 μg p66CH2CB were incubated with 30 μg decameric Prx1-Cys52Ser and different amounts of Trx1 (5/10/20 μg) in the presence of 3 mM EDTA and subjected to BN-PAGE.

p66Shc is known to generate H_2_O_2_ whereas peroxiredoxins exhibit weak but significant peroxidase activity. We therefore hypothized that Prx1 might degrade p66Shc-generated H_2_O_2_ and thereby inhibit p66Shc-dependent apoptosis. The other way round, p66Shc might inactivate Prx1 so that formed H_2_O_2_ does not get inactivated. In order to test this hypothesis, we analyzed the Prx1 forms in degrading H_2_O_2_ in a fluorescence-based ROS assay. While Prx1 WT decreased the fluorescence signal of 0.005 % H_2_O_2_ only slightly, the dimeric Prx1-Cys83Ser mutant significantly degraded H_2_O_2_ (Figure 3a). This result is consistent with published findings that dimeric Prx1 exhibits significant peroxidase activity, while the decameric form acts as a chaperone with decreased peroxidase activity [36,37]. As a control we generated a peroxidase-inactive Prx1 mutant by exchanging the peroxidasic cysteine residue Cys52 against Ser. As expected, this inactive mutant Prx1-Cys52Ser had no effect in the fluorescence-based ROS assay (Figure 3a).

Unfortunately, Prx1 peroxidase activity is inhibited by copper (data not shown), while H_2_O_2_-generation by p66Shc is copper-dependent [22,30]. Therefore, a measurement of the Prx1 effect on ROS-generation by p66CH2CB was not possible. However, we observed a dramatic increase in fluorescence at 0.005 % H_2_O_2_ in the presence of p66CH2CB without copper (Figure 3b), indicating an additional, copper-independent activity of p66CH2CB converting H_2_O_2_ to another ROS species. This copper-independent p66CH2CB activity was inhibited by Prx1 WT and the constitutively dimeric (peroxidasic) Prx1-Cys83Ser mutant (Figure 3b). Interestingly, the same effect was observed using the peroxidase-inactive Prx1-Cys52Ser mutant, strongly indicating that H_2_O_2_ degradation by Prx1 is not involved in this inhibitory effect. Our results indicate that p66CH2CB has an unexplored, copper-independent redox activity, which is inhibited by Prx1 independent of its peroxidasic activity.

We next tried to test the effect of the Prx1-dependent inhibition of p66CH2CB redox activity in an apoptosis induction assay using isolated mitochondria. Using a 4-fold excess of Prx1 over p66CH2CB to ensure sufficient complex formation, however, induced mitochondrial rupture even in absence of p66CH2CB (data not shown), making this assay incompatible with Prx1. Our results suggest to analyze the copper-independent p66Shc activity further in future studies employing other techniques, most importantly *in vivo* experiments, which promises exciting new insights into the physiological mechanisms of p66Shc-mediated redox signaling.

### Disulfide exchange between p66CH2CB and Prx1

We previously showed that the apoptosis inducing activity of p66CH2CB is activated by a reversible, oxidative dimer-tetramer transition, similar to regulation of Prx activity through disulfide-bridging. We therefore tested whether p66CH2CB and Prx1 influence the redox state of each other. The different states of p66CH2CB and Prx1 were detected by non-reducing SDS-PAGE: The denatured reduced forms run as monomers whereas the disulfide-linked forms behave as dimers (Figure 3c). After incubation of p66CH2CB with Prx1 WT, the reduced fraction of p66CH2CB is significantly increased whereas the reduced form of Prx1 WT decreases to a similar extent (Figure 3c). We therefore conclude that a reciprocal, thiol-based redox exchange reaction between oxidized, disulfide-bridged p66CH2CB and reduced Prx1 can occur. This redox reaction yields oxidized Prx1 and thus favors formation of dimeric, peroxidasic Prx1, consistent with our results of the BN-PAGE experiments (Figure 2b, 2c). At the same time, it yields reduced, dimeric p66CH2CB, which we previously showed to be an inactive form with respect to apoptosis induction. It thus appears that the p66Shc/Prx1 complex acts as an H_2_O_2_ degradation and sensing complex at low ROS levels, silencing the apoptotic function of p66Shc. At higher levels, Prx1 becomes overoxidized, peroxidase inactive, and stabilized in the decameric form, which should favour p66Shc release and tetramerization, and thus apoptosis induction.

To gain first insights into the dynamic composition and architecture of the p66Shc-based sensing complex, we tested whether the interaction of p66CH2CB with Trx influences complex formation with Prx1. We showed previously that Trx reduce and thus inactivate tetrameric p66CH2CB [30]. To avoid formation of mixed disulfides between Prx1 and Trx1 we used the Prx1-Cys52Ser active site mutant, which can still interact with p66CH2CB (see above). Adding Trx1 to p66CH2CB either before or during complex formation with Prx1-Cys52Ser did not prevent formation of the main complex band, but suppressed the weaker bands that appear to be caused by formation of further complexes through non-specific disulfide bonds (Figure 3d). It thus appears that Prx1 uses a different binding site on p66Shc than Trx. Furthermore, the major complex band appears to be the only significant form of the p66Shc/Prx1 complex, as the other forms are likely to be resolved *in vivo* by the Trx system.

## Discussion

p66Shc is a key player in stress sensing and the stress-induced mitochondrial apoptosis pathway. It acts as both, stress sensor and inducer of ROS formation, in a complex regulatory circuit [18,38]. Despite a wealth of *in vivo* observations, several molecular mechanisms connecting p66Shc and other players of this network remain to be fully understood, such as the exact mechanism of p66Shc-dependent ROS generation and the mechanism of ROS-dependent permeability transition. Such an understanding would also support efforts to exploit p66Shc as a therapeutic target. We reasoned that identifying novel interaction partners of the p66Shc-specific N-terminal domain could reveal insights into the regulation of its deadly activity. We could indeed show here that p66CH2CB interacts with the peroxiredoxin family of antioxidant enzymes that also contribute to signal transduction, e.g., of the p38 protein kinase [31,39]. In particular, our results show that p66Shc can interact with Prx1, and possibly with Prx3, in a physiological environment and *in vitro*. We observed a dominant enrichment of Prx1 in affinity experiments, and a much weaker signal from Prx3 as well as the characterized p66Shc interaction partner Hsp70 [28], highlighting the value of our mass spectrometry approach which allowed the identification of an unexpected and dominant but so far overlooked interaction partner.

**Figure 4.**
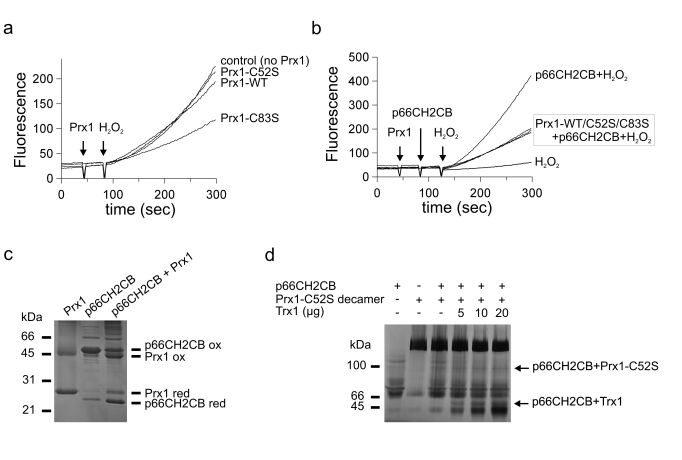
Working model for the p66Shc-centered regulatory network. Under conditions of lower stress, Trx keeps p66Shc reduced and dimeric. This p66Shc form interacts with dimeric Prx1, which also helps to keep p66Shc in its dimeric, apoptosis-inactive state and additionally degrades p66Shc-generated ROS. p66Shc in turn keeps Prx1 in its dimeric, peroxidase-active state. Excessive cellular stress, however, leads to disassembly of the p66Shc/Prx1 complex, formation of the decameric chaperone form of Prx1, and phosphorylation of p66Shc. The increased ROS-generating activity of phosphorylated p66Shc is compensated by complex formation with Pin1, which also leads to p66Shc translocation to the mitochondrial IMS. Here, tetrameric p66Shc is formed which finally induces apoptosis by initiating mitochondrial rupture through a still not fully understood mechanism, possibly involving its ROS-forming activity.

Prx can act as H_2_O_2_ scavengers, but many studies have shown that they are also major contributors to a complex ROS signaling system [31,40], most prominently in apoptosis initiation [41-43]. A Prx3 knock-down shows increased sensitivity to apoptosis inducing signals [44], and Prx1 is involved in signaling by the p38 kinase [39] and acts as tumor suppressor [45,46]. Prx1 is mainly found in the cytosol, although a recent determination of the mitochondrial proteome also identified Prx1 in this organelle [47]. The fact that Prx1 was more prominent in affinity experiment eluates than Prx3 even when mitochondrial lysates were used, which should contain more Prx3, indicates a pronounced preference of p66Shc for the Prx1 isoform. In fact, Prx3 is mainly located in the mitochondrial matrix, whereas the mitochondrial IMS, where p66Shc appears to induce apoptosis [22], is generally assumed to be strongly connected to the cytosol. Thus, Prx1 appears to be the major isoform encountered by p66Shc and seems to be bound preferentially, even in presence of other peroxiredoxins, indicating a pronounced level of specificity for this interaction. The fact that even a cysteine-lacking mutant of p66CH2CB could bind to Prx1 and disassembles its decamer form also reinforces the notion that the interaction is rather specific and does not resemble the redox interactions often encountered with thioredoxins, which appear to keep surface cysteines of cellular proteins reduced with little specificity. However, the observation that Prx1 reduces and thereby inactivates p66CH2CB indicates that Prx1 might functionally act like a Trx in this system. Protein/protein interactions have been described for Prx and are assumed to contribute to their function, in addition to H_2_O_2_ scavenging [31]. A regulatory role for an interaction with Prx1 has been described before for Ask1 [33], for example. In this system, the interaction rather than a redox reaction with Ask1 appears to be relevant for the apoptosis inhibiting effect of complex formation. It remains to be shown for the p66Shc/Prx1 complex whether purely forming the interaction, or the reciprocal reduction/oxidation, or both processes are relevant for its physiological function. At present we hypothize that both elements contribute to making this complex of two intrinsic ROS sensors [30,31] a highly regulated ROS sensing system (Figure 4): Complex formation keeps both players close in space, able to exchange information. The Prx, an efficient H_2_O_2_ scavenger at low concentrations [31], will protect cells from moderate oxidative stress, from external sources or formed by p66Shc. It will thereby keep p66Shc reduced and unable to induce apoptosis. The peroxidase activity of Prx1 will be inactivated through overoxidation, however, if excessive oxidative stress is encountered. This szenario would favor Prx1 decamerization, p66Shc release and oxidation as well as release of additional ROS by p66Shc now lacking its ROS-scavenging partner. This change will finally lead to the ultimate cellular stress response to overwhelming stress, apoptosis induction.

Prx show a versatile quaternary structure, apparently corresponding to different activities and or activation states [32,48]. Two interaction interfaces of a Prx monomer contribute to oligomerization of Prx of the A-type, which comprises mammalian Prx1 and Prx3 [32]. The first one is oriented parallel to the central β-sheet and mediates formation of a Prx1 dimer as its stable building block. A second one is oriented perpendicular to the central β-sheet and enables higher oligomerization of dimers. A "fully folded" conformation of the peroxidasic cysteine loop is observed in the reduced Prx1 form and stabilizes the perpendicular interface, promoting oligomerization. The "locally unfolded" conformation of the oxidized form destabilizes this interface and thus the decamer. The disassembling effect of p66CH2CB on Prx1 even in absence of the p66Shc Cys, and thus thiol redox reactions, suggest this interface as likely binding site for p66Shc with a direct communication contact to the Prx active site (Figure 4). Our results with added Trx indicate that an additional site on Prx can mediate further interactions, enabling formation of various complex forms, which can be reduced by Trx either through reduction or competition. The Prx1 binding site, however, does not serve as Trx binding site. The exact architecture of the p66Shc complexes formed with Prx1, however, will require future structural studies.

Another protein contributing to p66Shc regulation is Pin1 [24], a phosphoSer/Thr-Pro recognizing protein that can catalyze cis-trans isomerization within this motif. Pin1 has previously been implicated in aging processes and cellular stress responses [49], and the interaction with p66Shc is likely to be a mechanism contributing to these effects. Pin1 can bind to proteins and influence their stability, for example to p53 and cyclin D1 [49-52]. Pin1 has been reported to bind to phosphorylated p66Shc prior to transport into the IMS [24]. Our results indicate that Pin1 might act in the p66Shc system as a cytosolic inactivator, or stabilizer of a redox inactive p66Shc form (Figure 4). The exact nature of the p66CH2CB-dependent H_2_O_2_-metabolizing redox activity that can be silenced by Pin1 remains to be revealed. It might help to understand the discrepancy between p66Shc's apoptosis induction activity and its previously reported redox activity, H_2_O_2_ formation [22,30]. However, Pin1 was reported to be inactivated by oxidative stress [49,53], which would be consistent with an p66Shc-inhibiting function. Excessive cellular stress leading to Pin1 inactivation would promote the cytotoxic effect of p66Shc by allowing IMS localization and ROS formation.

Our results revealed novel regulatory effects and interactions within a p66Shc-centered signaling network. They should stimulate future studies on characterizing the coordination of these mechanisms *in vivo* and on the molecular architecture of the complexes formed by p66Shc. Such studies should lead to a further refined understanding of this complex signaling system, which should eventually enable development of drugs exploiting these mechanisms and interactions.

## Materials and methods

Cloning and protein purification. Cloning of full length p66Shc and of residues 1-150 (p66CH2CB) were described previously [30]. The gene for Prx1 was PCR-amplified from mouse colon cDNA and cloned into pET151/D-Topo (Invitrogen), resulting in a construct with an N-terminal 6x His-tag. Site-directed mutagenesis of p66CH2CB and Prx1 was done using the QuickChange protocol (Stratagene). Expression of WT proteins and variants was done as described for p66CH2CB [30]. Shortly, proteins were expressed in *E. coli* BL21(DE3)Rosetta2 cells cultured at 37 °C until the OD_600_ reached 0.6. Protein expression was induced by using 0.5 mM isopropyl β-D-thiogalactosid and cell culturing continued for 18 h at 20 °C. Harvested cells were disrupted using a French Press, and cell debris was removed by centrifugation (45 min at 18000 rpm, HFA22.50 rotor). Affinity chromatography was done with Talon resin (Clontech) (washing buffer 1: 20 mM Tris pH 7.8 + 500 mM NaCl; washing buffer 2: buffer A (20 mM Tris pH 7.8 + 150 mM NaCl) + 20 mM imidazole; elution buffer: buffer A + 100 mM imidazole). The proteins and their different oligomeric states were then further purified by size exclusion chromatography using a superose 12 column (GE Healthcare) in buffer A.

Non-reducing SDS-PAGE, blue-native (BN)-PAGE and 2D-PAGE. SDS-PAGE was performed according to Laemmli* et al.*[54] with a *T*=15 % separating gel. The samples were incubated with loading buffer devoid of reducing agents for 5 min at 95 °C. BN-PAGE according to Schaegger *et al.*[55] was performed using a separating gel consisting of a *T*=10 % and a *T*=14 % layer overlaid with a 5 % stacking gel. The samples were supplied with 10 % (v/v) Glycerol instead of sample buffer. In order to run a second, denaturing dimension of the natively separated complexes, the respective gel bands were cut out of the BN-gel and equilibrated  with 1 % (w/v) SDS and 1 % (v/v) β-mercaptoethanol first for 20 min at room temperature and second for 10 minutes at 50 °C. Subsequently, the gel pieces were washed extensively with distilled water and finally embedded into a SDS-PAGE stacking gel.

Mitochondria swelling assay and fluorescence-based ROS assay **. **Rat liver mitochondria were prepared freshly before use [30]. Swelling and rupture of mitochondria pretreated with 7 μM CaCl_2_ was monitored photometrically as a decrease of the OD_620_ as described previously [22]. The ROS-generating activity of p66CH2CB was measured using H_2_DDFDA (Invitrogen). For details see [22]. Changes in fluorescence (λ(ex)=498 nm, λ(em)=525 nm) of 10 μM H_2_DDFDA in 100 μl buffer A were recorded using a Perkin Elmer LS50B spectrofluorimeter.

Pull-down experiment and MudPIT analysis. 40 μl of Talon resin were saturated by incubation with 400 μg of His-tagged protein in the presence of 10 mM imidazole for 30 min at room temperature. Mitochondrial lysate (3 mg total mitochondrial protein) was added and incubated in the presence of 10 mM imidazole for 1 h at room temperature. The supernatant was removed and the affinity material first washed twice with washing buffer 1, and then twice with washing buffer 2. Finally, the His-tagged protein was eluted by addition of 30 μl elution buffer. Trypsin (Promega) was added to the eluate in a ratio of 1:100 and the proteins digested at 37 °C over night. Digestion was stopped with 0.25 % TFA and the peptide mixture analysed by MudPIT [34].
